# Death from Nitrous Oxide Gas

**Published:** 1877-06

**Authors:** S. Parsons Shaw

**Affiliations:** Manchester, England.


AMERICAN JOURNAL

OF

DENTAL SCIENCE.

Vol. XI. THIRD SERIES—JUNE, 1877. No. 2.

### ARTICLE I.

### *Death from Nitrous Oxide Gas.*

BY S. PARSONS SHAW, OF MANCHESTER, ENGLAND.

A melancholy case of death from the use of anæsthetic gas has occured in this city : and the verdict of the jury summoned by the coroner, declared that it had resulted from " syncope during the administration of nitrous oxide gas for the extraction of teeth, whilst laboring under fatty degeneration of the heart." Fearing that this statement may get into the American Journals and not unchallenged, I feel it a duty to make some explanations, as it must always be of the utmost importance that those of us who are daily administering this agent should have the fullest and most accurate information in a case attended with a fatal result.

The accounts which accompanied the report of the inquest in the Manchester papers, were substantially as follows :

Mr. George M. Harrison, aged fifty-three (53,) a well-known surgeon, formerly lecturer in one of our schools of medicine, went to a dentist to have some teeth extracted.

Mr. Harrison was much unnerved from excitement and loss of food and rest, and was very anxious to have sufficient gas to make him insensible. A quantity of gas was given, and an attempt made to extract the teeth, without success, he felt the pain. He then asked for more gas, held the mouth piece to his face for a time himself, inhaled vigorously, declined to comply with the request of the dentist, to hold up his hand as a sign that he had got enough, and directed that the inhalation be pushed until he snored. This was done, and two teeth were removed. As the patient did not recover, medical aid was summoned, when life was found to be extinct.

If nothing more was known of this unfortunate case, I apprehend that all who realize the almost insurmountable difficulties raised the moment the patient expresses the common wish that the administrator will "be sure to give him enough gas" would, upon the above facts alone, be strongly inclined to put an interrogation point after the verdict of coroner's jury.

It is natural that the profession should have a strong wish to know something more of this matter, and the lesson it teaches, as to the dangers attending the administration of the gas, than can be gathered from the verdict above quoted, and I am happy to be in a position to throw some light on the subject

The accomplished Manchester correspondent of the *Medical Times and Gazette*, in reviewing this case, concludes, from the facts above recited, that the jury were mistaken; and that death was not the result of heart disease, but probably of asphyxia. And since writing his communication, this gentleman has acquired from the records of the coroner's court, and by consultation with the gentlemen who made the post-mortem examination, the necessary facts to show that this was really the case. It should be here explained that this latter gentleman, although the one most competent to give evidence, was not called by the coroner as a witness, although in the court, but that the medical evidence was given second hand, by the surgeon who was first to see Mr. Harrison, and who consequently reported the case to the coroner.

From the facts gathered by the correspondent of the *Medical Times and Gazette*, and which he has generously allowed me to copy, *verbatim*, from his notes, I find that the post mortem appearances were as follows:

"The lungs on both sides were engorged with dark fluid blood; the right side of the heart was full of fluid blood, the left side was empty; there was a considerable quantity of fat beneath the skin, on the surface of the heart, in the omentum, and generally in the situation where fat is wholly deposited in corpulent persons. The muscular tissue of the heart was very slightly altered, in color and consistence, from incipient fatty degeneration. The liver was enlarged, pale and friable. The kidneys were full of blood, but otherwise healthy. The stomach contained a very small quantity of partially digested food, and a good deal of fœtid gas. The lining membrane of the trachea and bronchi was congested, some mucus was found in these tubes, but no blood or other foreign body. On removing the dura mater, a considerable quantity of cerebro-spinal fluid made its escape, the cornea of the lateral ventricles were dilated. The brain substance was healthy, and its vessels very full of dark fluid blood." Now, I would call especial attention to the fact that there was no more fat about the heart than we might expect in a patient in whom there was the tendency to obesity, revealed in its deposit in the places where it is in such cases wholly found; and also to what is the most important matter in this enquiry,—namely, that the muscular tissue of the heart was from fatty degeneration, but slightly altered in color and consistency. From this it appears so probable that the jury failed to comprehend the meaning of the evidence, and fell into an error not surprising, when directed by a non-medical coroner and confounded a *deposit of fat upon the heart*, *with fatty degeneration of the tissues of the heart itself*,—the latter being a serious pathological condition, while the former has but little pathological importance—that I think we may set aside that part of their verdict without further comment. Furthermore it may, I venture to assume, be just as safely decided that death occurred from apnœa—or, as more commonly but less accurately called asphyxia, for the general venous engorgement, the condition of the lungs and heart, the congested condition of the kidneys and the brain, and the serious effusion in the latter organ, all point to death from suffocation.

---

